# Case Report: Use of PET/CT to Guide Treatment in a Cat With Presentation Consistent With Hodgkin's-Like Lymphoma

**DOI:** 10.3389/fvets.2021.619264

**Published:** 2021-04-29

**Authors:** Carolyn L. Chen, Matthew R. Cook, Megan E. Brown, Sarah Lumbrezer-Johnson, Eric T. Hostnik, Janis M. Lapsley, Phillip Lerche, Vincent A. Wavreille, Maria I. Menendez, Michael V. Knopp, Sarah C. Linn, Christopher Premanandan, Laura E. Selmic

**Affiliations:** ^1^Veterinary Medical Center, The Ohio State University, Columbus, OH, United States; ^2^Department of Surgery, University of Zurich, Zurich, Switzerland; ^3^Department of Radiology, The Wright Center of Innovation in Biomedical Imaging, The Ohio State University Wexner Medical Center, Columbus, OH, United States; ^4^Department of Veterinary Biosciences, The Ohio State University, Columbus, OH, United States

**Keywords:** Hodgkin's, lymphoma, PET/CT, chemotherapy, fluorodeoxiglucose F18, lymphadenopathy

## Abstract

An 8-year-old male neutered Domestic Long Hair cat was presented for a cervical swelling that was suspected to be an enlarged left retropharyngeal lymph node. In the absence of other lymphadenopathy, this was initially suspected to be Hodgkin's-like lymphoma. A positron emission tomography–computed tomography (PET/CT) scan was performed using 2-deoxy-2-[^18^F]-fluorodeoxyglucose (^18^F-FDG) to assess for evidence of disease in other locations to guide treatment. Multifocal increased radiopharmaceutical uptake was identified, indicating disease in multiple organs. High-grade lymphoma was confirmed on tissue biopsy. As such, systemic cytotoxic chemotherapy was recommended instead of lymph node extirpation surgery. The cat received a modified CHOP chemotherapy protocol and attained a temporary partial remission. After 2 months of treatment, the cat stopped responding to chemotherapy and was eventually euthanized due to a relapse of disease and decreased quality of life. This case describes the utility of PET/CT to guide treatment in a cat with a presentation consistent with Hodgkin's-like lymphoma.

## Case Presentation

An 8-year-old male neutered Domestic Long Hair cat was presented for a rapidly growing left ventral cervical mass. The mass was first noted 3 weeks prior to presentation, and fine needle aspiration and cytology performed by the primary care veterinarian was suggestive of lymphoma. At the time of presentation, the cat had no clinical signs associated with the large mass. The owner reported that 7 months prior, there was a similar growth on the opposite side of the neck, which, after an inconclusive cytology, resolved with empirical antibiotic and steroid treatment. The cat had no other pertinent past medical history besides occasional upper respiratory signs from suspected feline herpesvirus infection.

Physical examination revealed a left ventral cervical mass (6.0 cm × 3.9 cm), which was suspected to be an enlarged medial retropharyngeal lymph node. No other enlarged lymph nodes were detected on palpation. A grade 2/6 left-sided systolic heart murmur was also noted. Initial complete blood count and chemistry panel revealed no significant findings, and Feline Leukemia Virus (FeLV)/Feline Immunodeficiency Virus (FIV) SNAP test were negative. Thoracic radiographs were unremarkable. An echocardiogram and abdominal ultrasound were recommended for further staging and to evaluate the extent of cardiac disease prior to general anesthesia but declined by the owner. Fine needle aspiration and cytology of the cervical mass revealed an expansion of large lymphocytes (~50%) with 50% small well-differentiated lymphocytes. There was also mild to moderate mixed inflammation (mild neutrophilic, moderate macrophagic). It is worthy to note that there was a lack of expansion of intermediate lymphocytes. These findings were concerning for lymphoma. Additional pertinent cytologic findings included mild plasma cell hyperplasia and mild to moderate mixed inflammation characterized by non-degenerate neutrophils and macrophages. Given the prolonged history of masses confined to the cervical region and cytology demonstrating persistence of a significant small lymphocyte population and mixed inflammation within the affected lymph node, Hodgkin's-like lymphoma was considered as a differential diagnosis in addition to high-grade lymphoma, despite the lack of characteristic Reed–Sternberg cells.

Cross-sectional imaging was recommended to stage and plan for potential surgical removal of the cervical mass. Case timeline is presented in Table 1. The next day, a computed tomography (CT) scan was performed under sedation (0.2 mg/kg butorphanol IV and 1.5 mg/kg alfaxalone IV), using a multidetector, 64-slice CT (GE Revolution EVO, GE Healthcare, Waukesha, WI), at 120 kVp in a transverse plane (1.25-mm slice thickness) with pre-contrast and post-contrast imaging with 10 ml (240 mgI/ml) of iohexol administered IV. The scan included the skull, cervical region, and the cranial thorax. The CT showed a 4.1 cm × 3.0 cm × 4.4 cm ovoid, hypoattenuating, heterogeneously and peripherally contrast enhancing soft tissue mass in the left ventral neck, causing rightward deviation of the trachea, esophagus, carotid arteries, and ventrolateral deviation of the left jugular vein. The mass was assumed to be the left medial retropharyngeal lymph node, as it was not seen otherwise. There was also enlargement, rounding, and contrast enhancement of the left superficial cervical lymph node (1.0 cm in diameter). Other findings from the CT included right caudal rhinitis and bilateral otitis media.

**Table 1:** Timeline of pertinent physical examination findings, diagnostics, and treatment.

**Visits**	**Diagnostic tests**	**Treatments**	**Prednisolone dose**	**Lymphadenopathy**
Week 0	Initial consultation, complete blood count, CT, PET/CT	None	None	L retropharyngeal: 6 cm × 3.9 cm L superficial cervical: mildly enlarged
Week 1	Complete blood count	Vincristine 0.6 mg/m^2^ IV Ondansetron 0.5 mg/kg IV	9 mg (1.9 mg/kg) PO q24	L retropharyngeal: 6 cm × 3.9 cm L superficial cervical: mildly enlarged
Week 2	Complete blood count, biopsy of L retropharyngeal lymph node	Vincristine 0.6 mg/m^2^ IV	9 mg (2.0 mg/kg) PO q24	L retropharyngeal: 3.2 cm × 1.5 cm L superficial cervical: mildly enlarged
Week 3	Complete blood count	Doxorubicin 1 mg/kg IV Maropitant 1 mg/kg IV Ondansetron 0.5 mg/kg IV	9 mg (2.1 mg/kg) PO q24	L retropharyngeal: 2.9 cm × 1.4 cm
Week 4	Complete blood count	Vinblastine 1.5 mg/m^2^ IV	5.4 mg (1.3 mg/kg) PO q24	L retropharyngeal: 2.2 cm × 1.1 cm
Week 5	Complete blood count	Cyclophosphamide 50 mg (185 mg/m^2^) PO	5.4 mg (1.2 mg/kg) PO q24	None
Week 6	Complete blood count	Vinblastine 1.5 mg/m^2^ IV	5.4 mg (1.3 mg/kg) PO q24	None
Week 7	Complete blood count, chemistry panel	Doxorubicin 1 mg/kg IV Maropitant 1 mg/kg IV	5.4 mg (1.3 mg/kg) PO q24	L retropharyngeal: 3.1 cm × 1.5 cm
Week 8	Complete blood count	Lomustine 10 mg (40 mg/m^2^) PO Maropitant 1 mg/kg IV	5.5 mg (1.4 mg/kg) PO q24	L retropharyngeal: 3.7 cm × 2.4 cm
Week 9	Complete blood count	None	5.5 mg (1.3 mg/kg) PO q24	L retropharyngeal: 3.25 cm × 2.5 cm L mandibular: 1.6 cm R mandibular: 1.7 cm
Week 10	Complete blood count, chemistry panel	Vincristine 0.6 mg/m^2^ IV Maropitant 1 mg/kg IV Ondansetron 0.5 mg/kg IV	5.5 mg (1.3 mg/kg) PO q24	L retropharyngeal: 4.0 cm × 2.5 cm R retropharyngeal: 1–1.5 cm L superficial cervical: 0.8–1 cm Bilateral popliteal: firm
4 days after previous visit	None	Euthanasia performed with Euthasol 5 mL IV	None	Physical examination not performed

A full-body positron emission tomography–computed tomography (PET/CT) was performed to assess the extent of local and distant disease the following week. PET/CT was recommended in addition to CT for the cat to obtain more sensitive staging information prior to surgical lymph node extirpation, especially due to the additional enlarged left superficial cervical lymph node noted on CT. The cat was premedicated with butorphanol (0.4 mg/kg IV); general anesthesia was induced with propofol (4 mg/kg IV) and maintained with isoflurane in 100% oxygen delivered via a circle anesthetic breathing system. The cat was placed in prone position. Blood glucose prior to 2-deoxy-2-[^18^F]-fluorodeoxyglucose (^18^F-FDG) injection was 102 mg/dl. A dose of 9.25 MBq/kg (0.25 mCi/kg IV) of ^18^F-FDG was administered to the cat. The list mode time-of-flight raw data were acquired continuously on the Gemini 64 TF with Astonish (Philips, Cleveland, Ohio) PET/CT system. Two-millimeter isotropic voxel data sets [288 × 288 matrix size using a 576 mm field of view (FOV)] and 180 s/bed were reconstructed using time-of-flight, three iterations, and 11 subsets. CT was acquired using the multi-slice system at 120 kVp and 150 mAs and reconstructed with a 4-mm slice thickness (512 × 512 matrix size using 600 mm FOV) for attenuation correction and coregistration. A dynamic acquisition was performed over the body region starting with the ^18^F-FDG administration followed by a whole-body PET at 75 min post injection. The Philips Intellispace Portal was used to generate the images for subsequent case review and semi-quantitative assessment.

**Figure 1:**
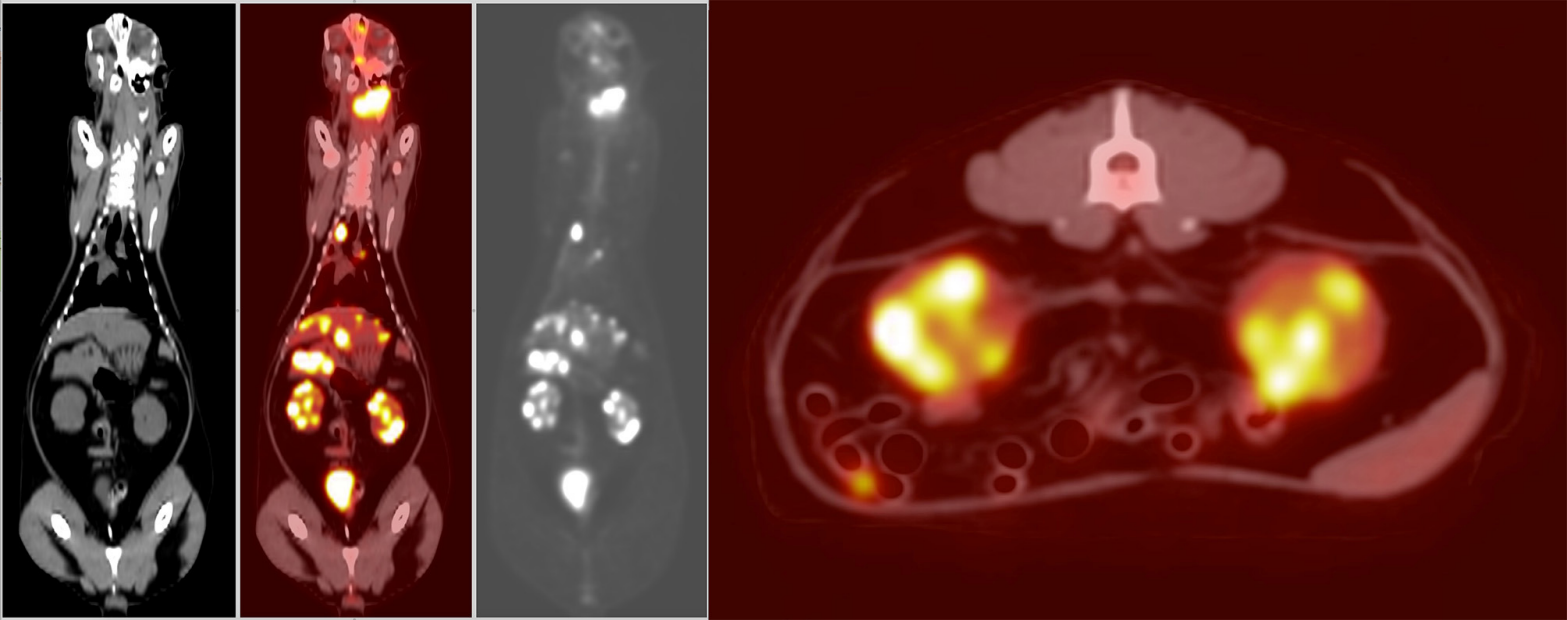
Dorsal plane whole-body CT, fused PET/CT, gray-scale PET, and axial plane fused PET/CT at the level of the kidneys (left to right). Showing increased ^18^F-FDG uptake in the region of the left medial retropharyngeal lymph node mass, as well as the nasal tissues, nasopharynx, right lung, mediastinum, liver, and both kidneys. Histopathology post-mortem confirmed large-cell high-grade lymphoma causing increased uptake in the kidneys. The increased ^18^F-FDG uptake in the urinary bladder is a normal finding. The increased ^18^F-FDG uptake in the small intestine is likely due to normal digestion.

PET/CT (Figure 1) showed multiple enlarged ^18^F-FDG-avid lymph nodes (SUVmax left medial retropharyngeal 31.0; right medial retropharyngeal 4.5; left caudal cervical 19.5) and nodal masses in the neck, in addition to multiple ^18^F-FDG-avid regions in both lungs (SUVmax 4.4 and 6.8) and the mediastinal lymph nodes (SUVmax 10.6 and 17.6). There were single soft tissue nodules with focal surrounding unstructured interstitial opacities in the caudal lungs bilaterally which were not appreciated on the thoracic radiographs performed prior. The small bowel had a ^18^F-FDG-avid soft tissue nodule. The liver had multiple hypoattenuating lesions that were ^18^F-FDG-avid (SUVmax 17.9), and both renal cortices had multiple foci of abnormally increased ^18^F-FDG uptake (SUVmax left kidney 16.7, right kidney 15.0). The spleen had no associated abnormal ^18^F-FDG uptake. The nasal cavity and nasopharynx had linear ^18^F-FDG activity with no definite CT abnormality (SUVmax 8.3). As a non-specific finding, it was interpreted as possibly inflammatory or infectious disease, or malignant involvement of the nasal soft tissues and adjacent osseous structures. There was mild ^18^F-FDG-avid partial opacification of the ethmoid sinuses (SUVmax 4.4) attributed to the underlying inflammatory sinus disease.

Given the multifocal nature of the disease found on PET/CT, surgery was not pursued, and chemotherapy was recommended. The cat was started on a CHOP-based protocol (Table 1) (1–5). A complete blood count was performed before every treatment to assess for myelosuppression and determine if the cat could receive chemotherapy. A physical examination before the first dose of chemotherapy confirmed a static 6.0 cm × 3.9 cm left retropharyngeal lymph node and mildly enlarged left superficial cervical lymph node. The cat received vincristine (0.6 mg/m^2^) IV and ondansetron (0.5 mg/kg) IV and went home on prednisolone (1.9 mg/kg) PO once daily. Maropitant, metronidazole, and ondansetron were prescribed on an as-needed basis.

The following week, physical examination revealed favorable response to the treatment; the left retropharyngeal lymph node was reduced in size (3.2 cm × 1.5 cm), although the left superficial cervical lymph node remained mildly enlarged. The cat was sedated (8 mcg/kg dexmedetomidine IV and 0.2 mg/kg butorphanol IV) for the administration of another dose of vincristine (0.6 mg/m^2^) IV, as well as an incisional biopsy of the left retropharyngeal lymph node, due to the lack of a definitive diagnosis from the previously performed fine needle aspirate. The biopsy was submitted for histopathologic evaluation at The Ohio State University.

**Figure 2:**
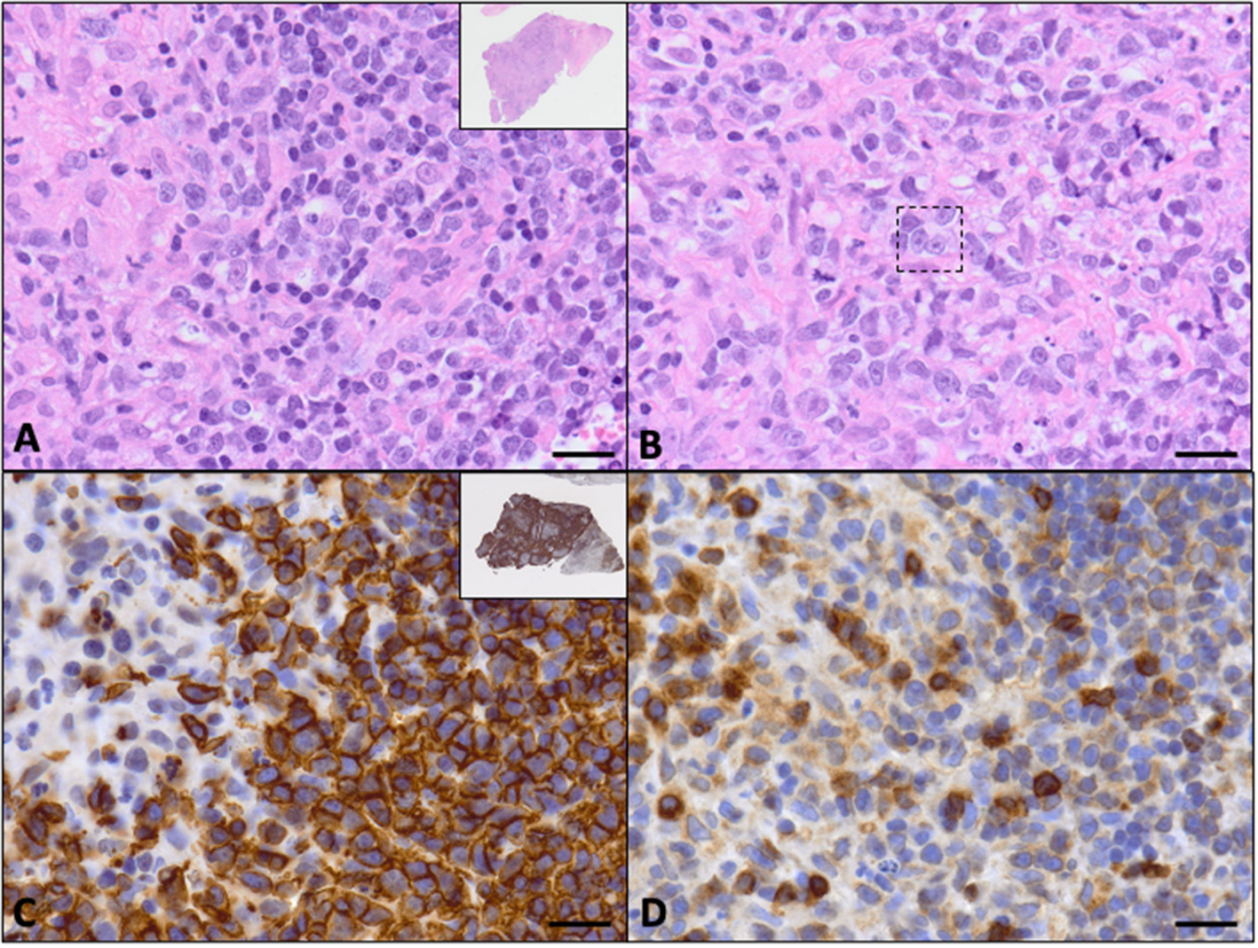
Evaluation of left retropharyngeal lymph node biopsy via H&E staining and immunohistochemistry (bar = 20 μM). **(A)** The neoplasm was composed of round neoplastic cells with little cytoplasm and nuclear pleomorphism. There are scattered neutrophils, macrophages, and nuclear debris (60×, H&E). The inset shows the entire biopsy specimen that was received (2×, H&E). **(B)** Rarely throughout the neoplasm, there were cells that appeared to have bilobed or binucleated nuclei with prominent eosinophilic nucleoli (within dotted line). These were suspect Reed–Sternberg cells and were not a prominent feature of the neoplasm (60×, H&E). **(C)** Neoplastic cells had strong membranous labeling with CD20, consistent with a B-cell neoplasm (60×, CD20 immunohistochemistry). The inset highlights the nodularity of the neoplasm with CD20+ cells surrounding foci of non-CD20, non-neoplastic cells (2×, CD20 immunohistochemistry). **(D)** There were scattered non-neoplastic lymphocytes that had strong membranous to cytoplasmic labeling for CD3 within the neoplastic population and within the non-neoplastic regions (60×, CD3 immunohistochemistry).

The submitted sections of lymph node had an invasive, non-encapsulated, highly cellular neoplasm composed of sheets resting on collagenous stroma. On low power, the neoplasm had vague and indistinct outlines of nodules composed of fibrous connective tissue (Figure 2A, inset). Neoplastic cells were round, had distinct cell borders, had variable amounts of eosinophilic to basophilic cytoplasm, and had large, round to irregular nuclei with vesicular to finely stippled chromatin and one large, eosinophilic, distinct nucleoli (Figure 2A). Rare (less than five) cells had nuclei that appear to be bilobed or binucleated with prominent eosinophilic nucleoli (suspect Reed–Sternberg cells) (Figure 2B). Occasionally, neoplastic cells had a moderate amount of vacuolated cytoplasm or had little to no cytoplasm with a clear halo surrounding. Mitoses were 15 in 10 high-powered (400 ×) fields. There were rare pockets of small lymphocytes throughout the tissue section. A moderate number of neutrophils and histiocytes were interspersed throughout the neoplasm with multifocal regions of necrosis characterized by eosinophilic homogenous material admixed with pyknotic cellular debris.

This biopsy was most consistent with a high-grade large-cell lymphoma. However, immunohistochemistry (IHC) was necessary to further characterize the neoplastic round cells. The neoplastic cells had strongly membranous labeling for CD20 (Figure 2C), and there was a distinct nodularity appreciated to the neoplasm (Figure 2C, inset). The neoplastic cells also had variably positive nuclear labeling for Pax5 (not pictured), but they were negative for CD3 (Figure 2D), supporting a diagnosis of large B-cell lymphoma.

Additional histopathology samples were then sent to the Leukocyte Antigen Biology Laboratory at the University of California at Davis for a second opinion. The described nodularity seen on H&E (Figure 2A, inset) and the CD20 stain (Figure 2C, inset) was further characterized with IHC with a histiocyte marker, Iba-1. The nodules were composed of histiocytes and enriched infiltrates of T cells. Peripheral to those nodular aggregates, there were dense accumulations of large neoplastic (CD20+) B cells as described previously. The neoplastic B cells had a high proliferative fraction (80–90% Ki-67 expression). PCR for antigen receptor rearrangements (PARR) was performed, and the neoplastic B cells were demonstrated to be clonal (KDEv, or Kappa deleting element variable gene). Due to the unique nodularity of the neoplasm, the interpretation of the neoplasm with IHC and PARR results was large B-cell lymphoma with inflammation. This diagnosis was favored over a histiocyte and T-cell-rich B-cell lymphoma (TCRBCL) because the neoplastic B-cell population was more abundant than what is typically seen with TCRBCL.

**Figure 3:**
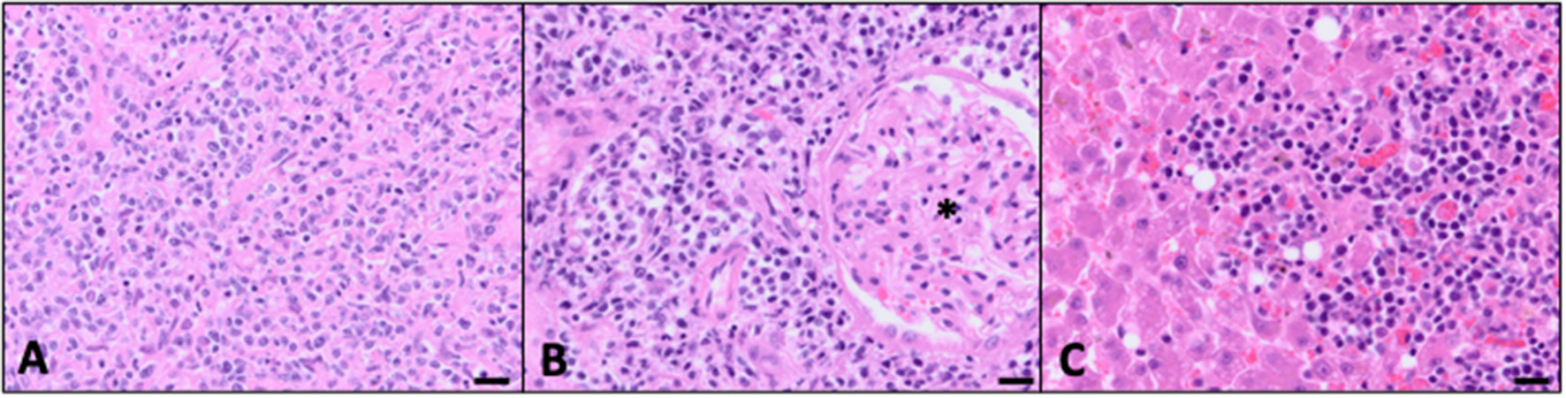
Evidence of multicentric disease confirmed at autopsy (bar = 20 μM). **(A)** Mandibular lymph node effaced with widespread neoplastic lymphocytes. Similar to the previous biopsy, there was an additional inflammatory infiltrate consistent with neutrophils, macrophages, and non-neoplastic lymphocytes (40×, H&E). **(B)** There were multifocal regions of neoplastic cells effacing parts of the renal cortical interstitium with loss of tubules. Many of the glomeruli (*) were left intact and surrounded by sheets of neoplastic cells (40×, H&E). **(C)** Neoplastic lymphocytes were clustered throughout the sections of liver corresponding to the tan to red masses seen grossly at autopsy. Additional microscopic foci of neoplastic cells extended into the sinusoids (40×, H&E).

The patient remained in a partial remission and was continued on a CHOP-based protocol for the following 6 weeks; vinblastine was substituted for vincristine because of a national drug shortage. However, progressive disease was noted on the eighth week, characterized by an increase in size of the left retropharyngeal lymph node (3.1 × 1.5 cm the previous week to 3.7 × 2.4 cm the current week). The patient was switched to a CCNU rescue protocol. Three weeks after the start of this rescue protocol, the patient was euthanized due to worsening lethargy, inappetence, and dyspnea, as well as progressive cervical lymphadenopathy. A limited necropsy was performed with identification of multifocal widespread infiltrative disease within the retropharyngeal and mandibular lymph nodes, kidneys, and liver. Histopathology of all of the tissues with identified lesions in PET/CT was not obtained. Similar to the hypoattenuated regions described in the PET/CT, the liver at necropsy had multifocal pale tan to red, firm masses ranging in size from 1 mm in diameter up to 5 cm × 3.5 cm × 3.0 cm. Both kidneys had similar appearing masses within the renal cortex. Histopathology confirmed large-cell high-grade lymphoma within both retropharyngeal and mandibular lymph nodes (Figure 3A), kidneys (Figure 3B), and liver (Figure 3C).

## Discussion

This case report described the use of a PET/CT for evaluating a cat with a clinical presentation consistent with Hodgkin's-like lymphoma. Currently, PET/CT is the standard of care for staging of Hodgkin's disease in humans (6), but such guidelines have not been established in veterinary medicine. In this case, we utilized PET/CT for staging of this patient, and the results contributed to both the diagnosis of multicentric high-grade B-cell lymphoma and treatment recommendations.

Hodgkin's-like lymphoma in veterinary medicine is a specific sub-type of nodal lymphoma that is histologically similar to Hodgkin's disease in humans (7). In humans, the most typical presentation is a painless, progressive enlargement of lymph nodes, most commonly in the neck and the supraclavicular region, with extra-nodal involvement being unusual (8). Cats with Hodgkin's-like lymphoma demonstrate similar presentations, with unilateral mandibular or cervical lymphadenopathy as the most common manifestation (9). This cat initially presented with a unilateral ventral cervical lymphadenopathy leading to the concern for Hodgkin's-like lymphoma.

Currently, there is no standard of treatment for feline Hodgkin's-like lymphoma. Previous retrospective studies have described a more systemic approach with chemotherapy (9), similar to treatment of Hodgkin's disease in humans with chemotherapy and radiation therapy combined (10). Some clinicians, however, also recommend surgical resection to control local disease (7). Therefore, appropriate workup and staging is crucial to ensure the correct avenue of treatment is pursued. In this cat's case, the clinician's choice of utilizing PET/CT as part of the evaluation of the cat proved critical in determining the appropriate treatment choice. With this functional imaging modality, ^18^F-FDG, a glucose analog, is used to map glucose metabolism in the body. Following cellular uptake, ^18^F-FDG becomes trapped in the cell and emits positrons, which subsequently create gamma photons that are detected by the PET scanner. This radionucleotide allows the detection of tumor cells, as these often use more glucose than normal cells creating regions on increased metabolic activity (11). A CT performed during the same study is fused with the PET imaging (11) and allows for precise anatomic localization of the increased metabolic activity. The PET/CT in this patient revealed suspect diffuse extra-nodal disease involvement, including ^18^F-FDG-avid regions in the lungs, nasal cavity, and nasopharynx that were not seen on prior staging. Since PET/CT allows for the detection of increased glucose analog utilization, identifying functional metabolism in neoplastic lymphocytes adds information to routine structural imaging modalities like ultrasound and conventional CT. The imaging findings, in addition to the cytology and histopathology of the grossly affected lymph node, indicated that surgical resection or radiation as the sole treatment was inappropriate.

Some limitations of this case include lack of further characterization of the lesions noted on the cat's workup. While extra-nodal disease was suspected on PET/CT imaging, none of the regions in the lungs, stomach, and nasal passages were sampled pre-mortem or post-mortem to confirm disease. Randall and colleagues describe the physiologic uptake of ^18^F-FDG in dogs and cats, which can be variable and mistaken for false positives (12). Cytological or histopathological examination of the affected regions could have better defined if there was disease or not. Similarly, only certain organs were evaluated on post-mortem histopathology, and no additional IHC was performed. Therefore, the extent of disease cannot be completely characterized; future studies should consider further detailing the extent of disease to correlate with imaging findings.

To the authors' knowledge, there are limited data regarding PET/CT for diagnosis and staging of neoplasia in veterinary medicine (11). Much of the data are limited to case reports or small case series, with rare canine case reports specific to lymphoma (13–15). Currently, there are limited to no reports discussing the use of PET/CT in feline lymphoma. This report establishes the utility of PET/CT as an additional diagnostic tool for feline multicentric lymphoma with a Hodgkin's-like presentation.

In this case, PET/CT provided identification of a multiorgan disease that was otherwise not detected in initial imaging workup. Thoracic radiographs did not reveal any abnormalities in the mediastinum and pulmonary parenchyma. One limitation of this study is that ultrasound was unable to be pursued to compare to the multiorgan involvement detected on PET/CT. Further research is necessary to assess sensitivity and specificity of ultrasound as compared to PET/CT for detection of lymphoma in veterinary medicine. Repeat PET/CT was also not pursued after chemotherapy treatment was initiated to confirm remission of the disease, although it is standard in human medicine to track response to the treatment. Further studies regarding this rare form of neoplasia in veterinary medicine should consider complete staging to confirm the diagnosis and monitor response to treatment and disease progression.

## Conclusion

The cat described in this report had cervical lymphadenopathy consistent with presentation of Hodgkin's-like lymphoma. However, further investigation with PET/CT identified extra-nodal sites of disease, and chemotherapy was recommended due to the multifocal nature of the disease. The PET/CT, cytology, and histopathology performed revealed an atypical presentation of multicentric high-grade B-cell lymphoma. A multi-agent chemotherapy protocol initially resulted in a partial remission, but the cat was ultimately euthanized due to progressive lymphadenopathy 3 months after start of treatment. PET/CT may have a role in the diagnosis and staging of feline cases with suspected Hodgkin's-like lymphoma.

## Data Availability Statement

The raw data supporting the conclusions of this article will be made available by the authors, without undue reservation.

## Author Contributions

CC wrote the manuscript. LS assisted in supervision of the clinical management of this case and contributed to conception of the case report. MB supervised the clinical management of this case. JL, VW, and PL assisted in supervision of the clinical management of this case. MC assisted in primary case management. MK and IM assisted in the imaging management of this case. EH and SL-J assisted in primary image reviewing. SL and CP assisted in pathology reviewing. All authors critically reviewed and approved the final version of the manuscript.

## Conflict of Interest

The authors declare that the research was conducted in the absence of any commercial or financial relationships that could be construed as a potential conflict of interest.
